# An Action-Based Psychosocial Group Intervention in Psychiatric Inpatient Care: A Pragmatic Add-On Study to Talk-Based Psychotherapy

**DOI:** 10.3390/healthcare14050687

**Published:** 2026-03-09

**Authors:** Jyu-Ming Hu, I-Fei Chen, Chun-Chu Lin, Li-Ting Huang, Nien-Hwa Lai, Ming-Wei Lin

**Affiliations:** 1 Institute of Health and Welfare Policy, National Yang Ming Chiao Tung University, Taipei 112, Taiwan; bgaa1818.md06@nycu.edu.tw; 2 Department of Psychiatry, Beitou Branch, Tri-Service General Hospital, National Defense Medical University, Taipei 112, Taiwan; p00436@mail.ndmctsgh.edu.tw (I.-F.C.); p00579@mail.ndmctsgh.edu.tw (C.-C.L.); p00729@mail.ndmctsgh.edu.tw (L.-T.H.); 3 Department of Psychology and Counseling, National Taipei University of Education, Taipei 106, Taiwan; annielai@tea.ntue.edu.tw; 4 Department of Medicine, National Defense Medical University, Taipei 114, Taiwan; 5 Institute of Health Policy and Management, National Taiwan University, Taipei 100, Taiwan; 6 Department of Psychiatry, Taichung Armed Forces General Hospital, Taichung 411, Taiwan

**Keywords:** action-based psychosocial intervention, psychodrama, psychiatric inpatient care, group psychotherapy, feasibility, multidisciplinary team, complexity-informed care

## Abstract

**Highlights:**

**What are the main findings?**

- An action-based psychosocial group intervention (psychodrama) was feasible to deliver as a pragmatic add-on to routine talk-based psychotherapy (TBP) in an acute inpatient setting, allowing for the exploratory observation of symptom trajectories under real-world constraints.
- While exploratory observations noted pre-to-post changes in internalizing and depressive symptom scores, between-group comparisons did not show statistical differences compared to TBP alone; however, the results support its practical viability as an additional clinical option.

**What are the implications of the main findings?**

- MDT co-facilitation of psychodrama broadens feasible psychosocial pathways, particularly for inpatients who may have difficulty sustaining exclusively verbal engagement during acute crises.
- Embedding action-based modalities alongside routine TBP may support MDT coordination and implementation of diversified, patient-centered psychiatric rehabilitation pathways within real-world ward workflows.

**Abstract:**

Background/Objectives: Psychiatric inpatient care emphasizes pharmacological stabilization, yet psychosocial interventions remain essential for addressing complex emotional, interpersonal, and behavioral needs. While talk-based psychotherapy (TBP) is standard inpatient psychosocial care, some patients face barriers to exclusively verbal engagement during acute crises. This study examined the feasibility and implementation of psychodrama as a pragmatic add-on to routine inpatient TBP under real-world ward conditions. Methods: A quasi-experimental pragmatic add-on design was used (*N* = 84). All participants received routine TBP; the experimental group (*n* = 47) additionally participated in psychodrama co-facilitated by a multidisciplinary team (MDT). Outcomes were assessed using the ASEBA-ASR. Non-parametric tests with effect sizes and 95% confidence intervals were used to evaluate pre–post symptom changes over four weeks, and between-group differences were assessed using change-score comparisons. Results: Both groups demonstrated significant within-group symptom reductions following the intervention. However, between-group comparisons of change scores showed no statistically significant differences (all *p* > 0.05), with small effect sizes and confidence intervals including zero. Conclusions: Psychodrama appears feasible to implement as an action-based psychosocial add-on in acute inpatient settings. Although statistical superiority over TBP alone was not demonstrated, the intervention allowed for the characterization of symptom trajectories under real-world constraints, suggesting that psychodrama may represent an action-based option to diversify psychosocial pathways within MDT-delivered care. Future adequately powered studies are needed to examine how action-based modalities may fit within multidimensional, complexity-informed inpatient care pathways.

## 1. Introduction

Psychiatric inpatient treatment is largely oriented toward pharmacological stabilization; however, medication alone is often insufficient to address the complex emotional, interpersonal, and behavioral needs of hospitalized patients [1,2]. Implementation-focused evidence indicates that, despite long-standing recognition of its clinical value, structured psychological therapies remain under-delivered in acute inpatient settings due to predictable barriers such as ward acuity, staffing constraints, and limited intervention infrastructure [1]. In parallel, contemporary global policy documents call for a system-level transition from a predominantly biomedical model toward integrated psychosocial services as core components of comprehensive mental health care, explicitly emphasizing service transformation and expansion of psychosocial care across the continuum, including inpatient services [2,3].

Across hospital settings, inpatient populations are increasingly recognized as presenting multidimensional complexity and layered vulnerabilities, for which single-domain symptom metrics may be insufficient [4]. Building on this complexity-informed lens, psychiatric hospitalization may likewise reflect intersecting psychosocial and functional vulnerabilities rather than isolated symptom episodes. Patients admitted to inpatient psychiatric services commonly present with affective and anxiety symptoms, trauma-related distress, and stress-related maladaptation. Large-scale evidence syntheses demonstrate that structured psychotherapies remain clinically relevant for key symptom domains: comprehensive meta-analytic evidence supports psychotherapy—particularly cognitive behavior therapy (CBT)—for depression [5]. Moreover, evidence from randomized trials and meta-analyses indicates that psychological interventions are efficacious for adjustment disorder and adult PTSD [6,7]. This escalating clinical complexity underscores the critical need for adjunctive, responsive psychosocial modalities that can be embedded within routine inpatient care to address diverse vulnerabilities beyond mere pharmacological stabilization.

Talk-based psychotherapy (TBP) is widely implemented in inpatient settings to support emotional processing, symptom management, and preparation for discharge. Group-based interventions, in particular, are commonly used in inpatient care due to scalability and their capacity to facilitate interpersonal learning and shared emotional regulation [8,9]. Nevertheless, engagement in purely verbal modalities can be difficult for some inpatients due to acute dysregulation, cognitive constraints, or defensive communication styles. Importantly, meta-analytic evidence suggests that matching patients’ treatment preferences with the interventions they receive is associated with lower dropout, improved treatment retention, and better clinical outcomes, highlighting the pragmatic value of offering more than one credible psychosocial pathway during hospitalization [10].

In East Asian cultural contexts, direct verbal disclosure of distress may be constrained by social norms valuing emotional restraint and face-saving (mianzi), which can further limit the reach of exclusively verbal models for some patients [11]. In Taiwan, psychodrama has developed across educational and applied settings as an action-based modality that facilitates experiential engagement and relational exploration, offering a culturally congruent route to access affect and interpersonal themes when linear narration is difficult [11]. Conceptually, action-based work may therefore function as a complementary pathway to TBP by enabling participation through embodied enactment and role interaction rather than relying solely on immediate verbal disclosure [12].

Psychodrama is commonly delivered as a structured group intervention that integrates enactment, role exploration, and guided sharing to support interpersonal learning and emotional processing [12]. Trauma-informed psychodrama further provides an implementation-ready rationale for acute wards by operationalizing safety, choice, collaboration, and empowerment through explicit role negotiation, paced intensity, and structured debriefing—features that are compatible with inpatient risk management priorities [13]. This structure also aligns with multidisciplinary team (MDT) delivery: psychiatrists, social workers, psychologists, and allied health professionals can share a procedural frame to coordinate preparation, containment, and post-session stabilization, thereby integrating action-based work as a pragmatic add-on that complements routine TBP rather than displacing it [13].

Recent evidence syntheses support the potential clinical utility of psychodrama and broader drama-based therapies across diverse populations. A meta-analysis focusing on Chinese samples reported that psychodrama is associated with improvements in mental health outcomes based largely on controlled study designs, suggesting relevance for Chinese cultural contexts [14]. In addition, an APA-journal meta-analysis of drama-based therapies reported beneficial effects across controlled studies, suggesting that action-oriented and dramaturgical elements may contribute to therapeutic effects beyond purely expressive engagement [15]. Inpatient-relevant evidence further indicates that psychodrama has been associated with pre–post reductions in PTSD and depressive symptoms in inpatient substance use treatment settings, supporting its potential usefulness under real-world ward constraints [16]. Together, these findings justify evaluating psychodrama as an evidence-informed adjunct within inpatient care.

Despite promising findings, the psychodrama evidence base remains limited by heterogeneity in design quality and the relative scarcity of rigorous comparative studies directly evaluating psychodrama against TBP within inpatient psychiatric settings. Controlled-trial syntheses suggest that psychodrama group therapy can be operationalized as a structured intervention with measurable outcomes, supporting its status beyond informal activity-based programming [17]. Specifically, mechanism-oriented scholarship suggests that concretization—the process of transforming diffuse internal experiences into tangible representations through enactment—serves as a primary mechanism of change [18]. In addition, practical elements such as sociometric processes have been described as facilitating rapid relational mapping and group cohesion [19], a principle that may be particularly useful for maintaining therapeutic direction in semi-open inpatient groups. More broadly, contemporary scoping reviews emphasize the importance of linking these therapeutic factors to observable change mechanisms in creative arts therapies [20]. These inpatient delivery constraints motivate the use of pragmatic designs that evaluate interventions under routine conditions rather than idealized delivery contexts. The shift toward ‘pragmatic precision psychiatry’ encourages the evaluation of interventions within routine care to optimize treatment selection for diverse clinical populations, thereby bridging the gap between ideal-world efficacy and real-world clinical effectiveness [21]. In line with global calls for service transformation toward integrated psychosocial care [2,3], a pragmatic additive design is particularly suitable for testing psychodrama as an add-on to routine inpatient TBP. Furthermore, recent international calls to action emphasize that improving the quality and accessibility of inpatient group therapy is a critical priority for modern mental health services, necessitating studies that demonstrate how specific modalities can be integrated into high-acuity wards [22].

From a patient-centered perspective, meta-analytic evidence suggests that aligning interventions with patient preferences—such as offering meaningful treatment options beyond purely verbal models—is associated with improved clinical outcomes, higher satisfaction, and reduced treatment dropout [23]. Therefore, evaluating add-on modalities like psychodrama informs the development of evidence-based care pathways that respect and empower patient choice in acute care settings [24].

Importantly, this study is situated in a distinctive implementation context within Taiwan’s hospital-based mental health system: psychodrama is delivered as an MDT co-facilitated, action-based group intervention integrated into routine inpatient care. Accordingly, this study conducts a pragmatic add-on evaluation of psychodrama implemented under routine inpatient conditions in Taiwan. Using a pragmatic additive design, we examine an action-based psychodrama program delivered alongside routine inpatient talk-based psychotherapy (TBP). We assess pre–post changes in depression and ASEBA Adult Self-Report (ASR) problem domains to describe symptom trajectories under real-world ward constraints. In addition, we explore between-group differences in change scores between the psychodrama add-on group and the TBP-only group as an exploratory comparison, without prespecifying superiority, equivalence, or non-inferiority hypotheses. Collectively, the findings are intended to inform feasibility and hypothesis generation for future controlled studies incorporating broader multidimensional outcomes relevant to complexity-informed inpatient care (e.g., engagement, functioning, and discharge readiness).

## 2. Materials and Methods

### 2.1. Participants

Participants were recruited from the psychiatric inpatient unit of the military-affiliated psychiatric hospital. The inclusion criteria encompassed hospitalized individuals diagnosed with depressive disorders (ICD-10: F32, F33, F34) and stress-related disorders (F43), as confirmed by attending psychiatrists. To be eligible, patients were required to attend at least one therapy session, either psychodrama or talk-based psychotherapy, during hospitalization, a pragmatic criterion chosen to capture the naturalistic turnover and variable length of stay inherent in acute ward settings. Exclusion criteria included diagnoses of schizophrenia, bipolar disorder, intellectual disability, or substance use disorders, as these conditions typically require distinct, highly specialized stabilization protocols (e.g., acute psychosis containment or substance withdrawal) that differ fundamentally from the affective and stress-related pathways targeted in this initial pragmatic trial. Eligible participants received a face-to-face explanation of the study, and written informed consent was obtained prior to enrollment.

A total of 91 patients were initially enrolled, including 50 in the psychodrama group and 41 in the talk-based psychotherapy group. Seven participants (three from the psychodrama group and four from the talk-based psychotherapy group) were excluded from the final analysis due to incomplete questionnaire data. The final analytic sample therefore consisted of 84 participants.

### 2.2. Study Design

This study employed a quasi-experimental pragmatic add-on design comparing TBP plus psychodrama versus TBP alone. Both intervention groups received routine pharmacological treatment as part of standard inpatient care. Due to the logistical constraints of ward management and clinical scheduling, randomization at the individual level was not feasible. Therefore, participants were allocated to either the psychodrama group or the talk-based psychotherapy group based on clinical availability and group scheduling. This study employed a group comparison approach conducted within two psychiatric inpatient wards (Ward A and Ward B). Participants were admitted to Ward A or Ward B according to routine hospital admission procedures rather than research allocation.

Importantly, because the study was conducted during the COVID-19 pandemic, strict hospital infection control protocols prohibited physical interaction or movement between wards. This provided an ethically sound framework for the ward-based design, as it allowed for the evaluation of the add-on intervention without compromising infection control mandates, while simultaneously providing a natural barrier against treatment contamination between cohorts. Ward A offered psychodrama group sessions in addition to routine talk-based psychotherapy (TBP), whereas Ward B provided standard talk-based psychotherapy only. Patients participated in the therapy program available in the ward to which they were admitted.

**Psychodrama Group** (*n* = 47): Received TBP supplemented with psychodrama (Action-Based Psychosocial Group Intervention).

**Comparison Group** (*n* = 37): Received TBP alone.

### 2.3. Interventions

#### 2.3.1. Talk-Based Psychotherapy (TBP)

This served as the standard of care for both groups (and was the sole psychosocial intervention for the control group). The talk-based psychotherapy group met twice weekly for 90 min sessions, following a structured discussion format that emphasized cognitive and emotional processing through verbal exchange. These interventions were delivered by licensed mental health professionals as part of the ward’s routine schedule.

#### 2.3.2. Action-Based Psychosocial Group Intervention (Psychodrama)

In addition to routine TBP, the experimental group participated in psychodrama group sessions twice weekly, with each session lasting approximately 90 min over a four-week period. These sessions were facilitated by a multidisciplinary therapeutic team (MDT), including psychiatrists, clinical psychologists, social workers, and occupational therapists. To ensure intervention fidelity, the facilitation team received regular supervision from a TEP-certified psychodrama trainer. Each session followed a consistent triadic structure (warm-up, enactment, and sharing) and was documented via routine clinical session records. However, sessions were not independently rated for fidelity via standardized checklists, which is addressed as a limitation. Additionally, while the study was conducted during the COVID-19 pandemic, strict hospital infection control protocols restricted and limited movement and direct interaction between wards, which likely minimized or substantially reduced cross-ward contamination; however, residual contamination cannot be fully excluded.

### 2.4. Measures

Psychological outcomes were assessed using the Achenbach System of Empirically Based Assessment—Adult Self-Report (ASEBA-ASR), a standardized instrument validated for diverse clinical populations. Use of the ASR was supported by psychometric evaluations of the Chinese version, demonstrating robust internal consistency and validity for indexing psychological symptoms in Chinese adult samples [25]. Primary outcome domains included depression (indexed using the ASR DSM-oriented Depressive Problems scale), internalizing problems, externalizing problems, and total problems.

### 2.5. Statistical Analysis

Descriptive statistics were used to summarize participant characteristics. Between-group comparisons of categorical variables (e.g., sex, education level) were conducted using chi-squared tests, while continuous variables (e.g., age, pre-treatment symptom scores) were analyzed using the Mann–Whitney U test due to the relatively small sample size and non-normal distribution of several variables. Within-group treatment effects were examined using the Wilcoxon signed-rank test to assess pre-to-post changes. To compare treatment outcomes between groups, Mann–Whitney U tests were performed to analyze differences in symptom reduction across conditions. Rank-based effect sizes (r) were calculated for non-parametric tests, and standardized effect sizes (Cohen’s d) with 95% confidence intervals were calculated for between-group comparisons. To control for multiple testing across the four outcome variables, Bonferroni correction was applied. A post hoc power analysis was conducted using G*Power (version 3.1.9.7) based on the observed sample size and α = 0.05 (two-tailed). Statistical significance was set at *p* < 0.05. All statistical analyses were conducted using MedCalc^®^ version 23.1.6.

## 3. Results

### 3.1. Participant Characteristics

**Figure 1:**
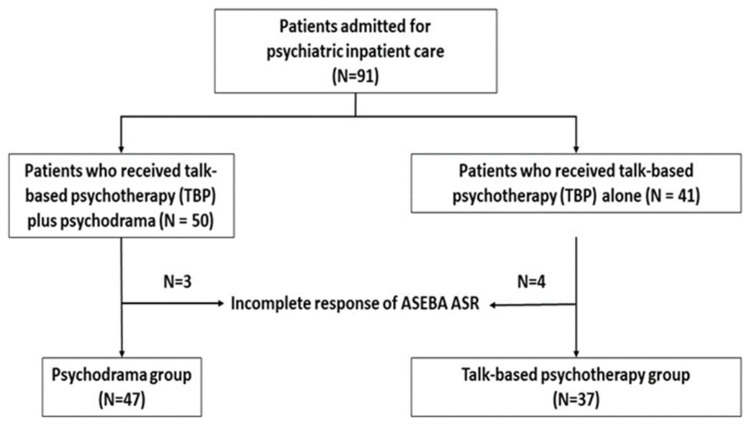
CONSORT flow diagram of participant recruitment and allocation.

A total of 84 patients completed the study. Table 1 presents the sociodemographic characteristics and baseline symptom scores of participants in the psychodrama and the talk-based psychotherapy group. There were no significant differences between the Psychodrama group (*n* = 47) and the Talk-Based Psychotherapy group (*n* = 37) in age, gender, education level, or baseline symptom severity (all *p* > 0.05). Given the non-randomized design, these findings suggest generally similar baseline characteristics. The sample was predominantly male (~83%) with a mean age of approximately 23 years. The participant flow is illustrated in Figure 1.

### 3.2. Within-Group Analysis

**Figure 2:**
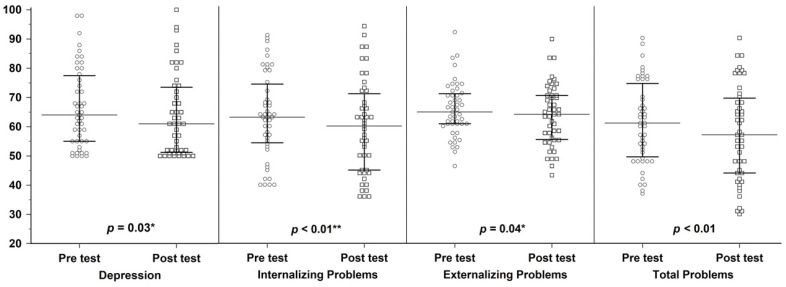
Reduction in Depression, Internalizing Problems, Externalizing Problems, and Total Problems scores in the psychodrama group. (* *p* < 0.05; ** *p* < 0.01).

Participants in the psychodrama group demonstrated significant within-group improvements across all measured domains following the intervention. Specifically, statistically significant reductions were observed in Depression (z = −2.16, *p* = 0.03), Internalizing Problems (z = −2.75, *p* < 0.01), Externalizing Problems (z = −2.01, *p* = 0.04), and Total Problems (z = −3.10, *p* < 0.01) (Figure 2). Similar within-group improvements were also observed in the talk-based Psychotherapy group, with significant reductions in Depression (z = −3.23, *p* < 0.01), Internalizing Problems (z = −3.35, *p* < 0.001), Externalizing Problems (z = −2.59, *p* < 0.01), and Total Problems (z = −3.29, *p* < 0.01) (Table 2). After Bonferroni correction for multiple comparisons across the four outcome measures, statistically significant within-group improvements in the psychodrama group remained for Internalizing Problems and Total Problems, whereas all outcomes remained statistically significant in the talk-based psychotherapy group. Although the magnitude of change was modest (~0.24 SD), the reduction in Internalizing Problems corresponded to a shift in the mean T-score from the clinical range (T ≥ 63) to below the borderline threshold (T = 60–63). This threshold shift may suggest symptom stabilization over the short duration of acute treatment, but clinical meaningfulness cannot be firmly established without validated MCID benchmarks and clinically anchored outcomes.

### 3.3. Between-Group Analysis

**Figure 3:**
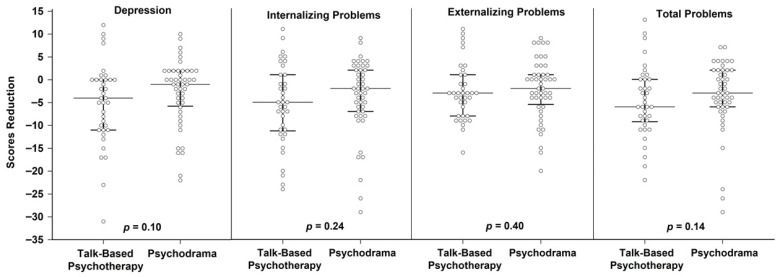
Comparison of change scores in Depression, Internalizing Problems, Externalizing Problems, and Total Problems between the psychodrama group and talk-based group psychotherapy.

When comparing pre-to-post treatment changes between the two groups, there were no statistically significant differences between the psychodrama and talk-based psychotherapy groups. Reductions in Depression (*p* = 0.10, d = 0.30, 95% CI −0.13 to 0.73), Internalizing Problems (*p* = 0.24, d = 0.21, 95% CI −0.22 to 0.65), Externalizing Problems (*p* = 0.40, d = 0.08, 95% CI −0.35 to 0.52), and Total Problems (*p* = 0.14, d = 0.16, 95% CI −0.28 to 0.59) did not differ significantly between the psychodrama and talk-based psychotherapy groups (Table 2; Figure 3). The observed effect sizes were small, and the confidence intervals included zero for all outcomes, indicating no statistically reliable between-group differences. Therefore, no statistically significant difference in treatment effects between the two conditions was detected in the present sample. A post hoc power analysis indicated that the statistical power for detecting between-group differences was 0.61, suggesting that the study may have been underpowered to detect small between-group differences.

## 4. Discussion

This pragmatic quasi-experimental study examined the feasibility and clinical outcome signals of implementing psychodrama group psychotherapy as an add-on to talk-based psychotherapy (TBP) in a psychiatric inpatient setting. Although both groups experienced within-group symptom reductions over the four-week intervention, between-group comparisons of change scores did not demonstrate statistical superiority of the add-on approach. Accordingly, these findings should be interpreted primarily as feasibility evidence regarding the operationalization and integration of action-based MDT programs within the high-acuity constraints of routine ward workflows, rather than as confirmatory evidence of comparative therapeutic efficacy. While psychodrama may be considered as an action-based option to broaden psychosocial modalities for inpatients who experience barriers to exclusively verbal engagement, this remains a preliminary observation. The study’s primary value lies in describing a feasible implementation model that balances therapeutic complexity with the logistical demands of acute psychiatric care.

The within-group reductions in depression and internalizing symptoms observed in this study are consistent with contemporary findings [11,12], which suggest that action-oriented interventions foster emotional expression, cognitive restructuring, and behavioral activation. While traditional TBP provides essential cognitive grounding, psychodrama’s emphasis on enactment and role-playing facilitates emotional catharsis by allowing patients to externalize distressing experiences in a safe, “as-if” therapeutic environment [11,12]. Furthermore, the reduction in internalizing distress was measured using the Adult Self-Report (ASR), which recent psychometric evaluations have confirmed as a robust and valid tool for indexing psychological symptoms in Chinese adult populations [25]. By leveraging structured group modalities, our intervention balanced therapeutic flexibility with clear procedural frames—a design increasingly recognized as essential for managing clinical complexity and ensuring safety in high-acuity, high-turnover ward settings [26].

Our findings also highlight the potential utility of psychodrama in addressing externalizing problems. Participants in the experimental group demonstrated initial within-group reductions in externalizing distress (though this did not survive conservative multiple-comparison corrections), supporting previous evidence that drama-based therapies benefit patients with difficulty regulating emotions and behaviors [14,27]. The interactive and embodied nature of psychodrama facilitates greater self-awareness and impulse control through experiential learning [12].

The lack of statistically significant between-group differences may be attributed to common therapeutic factors inherent in group-based interventions, such as cohesion, social support, and interpersonal alliance [8,9]. Although between-group differences were not statistically significant, the core therapeutic mechanisms differ conceptually: psychodrama utilizes concretization to transform diffuse internal experiences into tangible representations [18], whereas TBP focuses on verbal processing and cognitive reflection. Given the absence of statistically significant between-group differences, interpretations regarding specific therapeutic mechanisms (e.g., concretization, role reversal) should be considered exploratory hypotheses rather than confirmatory explanations of intervention-specific effects.

Crucially, the interpretation of these findings must be situated within the evolving landscape of inpatient psychiatric care. Across hospital settings, inpatient populations are increasingly recognized as presenting multidimensional complexity [4]. Viewed through this complexity-informed lens, contemporary psychiatric hospitalization may likewise reflect a high psychiatric burden and layered psychosocial vulnerabilities. Consequently, evaluating psychodrama solely on short-term symptom reduction metrics may not capture its full clinical utility. This aligns with the updated Medical Research Council (MRC) framework for complex interventions, which emphasizes that traditional effectiveness metrics often fail to capture the broader system-level value and feasibility of complex psychosocial programs [28]. In this view, feasibility, implementation processes, and system-level outcomes are integral to evaluating complex inpatient psychosocial programs, alongside symptom change. Beyond individual clinical outcomes, the multidisciplinary team (MDT) co-facilitation model employed here has broader implications for hospital organizational culture. Transforming the treatment model from isolated provider-patient interactions into a dynamic, team-based approach may support interdisciplinary coordination and shared clinical language [29]. However, team- and system-level outcomes (e.g., staff well-being, ward climate) were not explicitly measured in the present study and should be examined in future work.

Several limitations warrant consideration. First, the pragmatic, semi-open group design with voluntary participation precluded systematic individual attendance tracking. Consequently, we could not examine dose–response relationships or causally isolate the observed improvements from natural stabilization, pharmacological effects, or nonspecific ‘dosage’ effects (i.e., increased therapeutic contact time). Second, while sessions followed a structured triadic format under expert supervision, they were not independently rated for fidelity using standardized checklists. Third, although ward-based allocation and COVID-19 infection control protocols likely minimized cross-ward interaction, the risk of residual treatment contamination in a real-world ward milieu cannot be fully excluded. Fourth, a fundamental limitation is the disjuncture between our complexity-informed conceptual framing—emphasizing action-based engagement and cultural fit—and our unidimensional measurement. The ASR was administered according to standard instructions; however, because it is standardized with a longer recall window (typically six months), it may be less sensitive to rapid, state-level symptom changes in acute contexts. Furthermore, the study relied solely on self-report data and omitted multi-informant measures (e.g., clinician ratings) and critical process-oriented or functional outcomes—such as therapeutic alliance, group cohesion, cultural perceived fit (e.g., mianzi), and discharge readiness. The absence of these indicators restricts our ability to directly test the hypothesized mechanisms through which psychodrama might bypass verbal engagement barriers. Fifth, the demographic composition (predominantly young military personnel) strongly reflects the specific catchment area of the military-affiliated psychiatric hospital, limiting generalizability to broader civilian populations. Finally, a post hoc sensitivity contextualization indicated limited power (power = 0.61) to detect small between-group effects. We acknowledge that post hoc analysis is not a substitute for a priori planning and does not remedy the study’s design limitations; rather, it serves as a retrospective diagnostic of the underpowered status of this pragmatic trial. Importantly, the absence of statistically significant differences does not indicate equivalence. Future research should utilize adequately powered randomized designs with attention-matched active controls and comprehensive outcome batteries—including symptom-specific instruments (e.g., BDI, BAI), functional outcomes (e.g., therapeutic alliance, discharge readiness), and objective systemic indicators (e.g., readmission rates, emergency department utilization)—to more robustly evaluate these preliminary clinical signals.

## 5. Conclusions

In conclusion, this study indicates that psychodrama is feasible to implement as an action-based psychosocial group intervention when delivered as a pragmatic add-on to routine talk-based psychotherapy (TBP) in acute psychiatric inpatient care. Although exploratory between-group comparisons did not show statistically significant differences compared to TBP alone, the add-on condition allowed characterization of symptom trajectories under real-world ward constraints. Overall, these findings suggest that psychodrama may represent an action-based option to diversify patient-centered care pathways, particularly for inpatients who face barriers to exclusively verbal engagement. To transition from these preliminary observations to evidence-based recommendations, future research should employ adequately powered randomized designs with attention-matched active control conditions, preference-based trial frameworks, and comprehensive implementation evaluations incorporating multidimensional outcome batteries.

## Figures and Tables

**Table 1:** Sociodemographic Characteristics and Pre-Test Scores Between the Psychodrama Group and the Talk-Based Psychotherapy Group.

	Psychodrama (*n* = 47)		Talk-Based Psychotherapy (*n* = 37)		*p*-Value
	*n*/Mean	(%)/SD	*n*/Mean	(%)/SD	
Sex					0.63
Male	40	85.11%	30	81.08%	
Female	7	14.89%	7	18.92%	
Age	23.62	4.38	23.14	4.37	0.62
Education					0.43
High school and below	20	42.55%	21	56.76%	
College and above	27	57.45%	16	43.24%	
Depression	66.62	13.69	68.00	12.28	0.63
Internalizing problems	63.51	14.50	65.35	12.88	0.55
Externalizing Problems	57.00	11.52	55.41	10.64	0.52
Total Problems	61.19	13.87	61.57	12.19	0.90

**Table 2:** Pre- and post-test outcome scores in the psychodrama and talk-based psychotherapy groups with within- and between-group comparisons.

	Psychodrama (*n* = 47)			Talk-Based Psychotherapy (*n* = 37)			Between Group *p*-Value (Δ)	Cohen’s d (95% CI)
	Pre-Test (Mean/SD)	Post-Test (Mean/SD)	Within Group *p*-Value (Δ)	Pre-Test (Mean/SD)	Post-Test (Mean/SD)	Within Group *p*-Value (Δ)		
Depression							0.10	0.30 (−0.13–0.73)
	66.62/13.69	63.81/14.09	0.03 *	68.00/12.28	62.78/12.16	<0.01 **		
Internalizing Problems							0.24	0.21 (−0.22–0.65)
	63.51/14.50	59.70/15.95	<0.01 **	65.35/12.88	59.73/14.94	<0.001 ***		
Externalizing Problems							0.40	0.08 (−0.35–0.52)
	57.00/11.52	54.83/13.01	0.04 *	55.41/10.64	52.73/12.86	<0.01 **		
Total Problems							0.14	0.16 (−0.28–0.59)
	61.19/13.87	57.57/16.31	<0.01 **	61.57/12.19	56.76/14.54	<0.01 **		

* *p* < 0.05; ** *p* < 0.01; *** *p* < 0.001.

## Data Availability

The data presented in this study are available on request from the corresponding author due to privacy restrictions.
